# Endovascular Treatment and Outcomes for Femoropopliteal In-Stent Restenosis: Insights from the XLPAD Registry

**DOI:** 10.1155/2022/5935039

**Published:** 2022-07-15

**Authors:** Michael H. Vu, Glaiza-Mae Sande-Docor, Yulun Liu, Shirling Tsai, Mitul Patel, Chris Metzger, Mehdi H. Shishehbor, Emmanouil S. Brilakis, Nicolas W. Shammas, Peter Monteleone, Subhash Banerjee

**Affiliations:** ^1^Methodist Health System of Dallas, Dallas, TX, USA; ^2^University of Texas Southwestern Medical Center, Dallas, TX, USA; ^3^Veterans Affairs North Texas Health Care System, Dallas, TX, USA; ^4^University of California San Diego Sulpizio Cardiovascular Center, La Jolla, San Diego, CA, USA; ^5^Ballad Health/Holston Valley Medical Center, Kingsport, TN, USA; ^6^Case Western Reserve University and Harrington Heart and Vascular Institute, University Hospitals, Cleveland, OH, USA; ^7^Minneapolis Heart Institute, Minneapolis, MN, USA; ^8^UnityPoint Health-Trinity Bettendorf, Bettendorf, IA, USA; ^9^Ascension Seton Medical Center Austin, Austin, TX, USA

## Abstract

**Background:**

There is limited “real-world” evidence examining treatment modalities and outcomes in patients with symptomatic peripheral arterial disease undergoing endovascular treatment of femoropopliteal (FP) in-stent restenosis (ISR).

**Materials and Methods:**

We compared outcomes in 2,895 patients from the XLPAD registry (NCT01904851) between 2006 and 2019 treated for FP ISR (*n* = 347) and non-ISR (*n* = 2,548) lesions. Primary endpoint included major adverse limb events (MALE) at 1 year, a composite of all-cause death, target limb repeat revascularization, or major amputation.

**Results:**

ISR patients were more frequently on antiplatelet (94.5% vs 89.4%, *p*=0.007) and statin (68.9% vs 60.3%, *p*=0.003) therapies. Lesion length was similar (ISR: 145 ± 99 mm vs. non-ISR: 142 ± 99 mm, *p*=0.55). Fewer treated ISR lesions were chronic total occlusions (47.3% vs. 53.7%, *p*=0.02) and severely calcified (22.4% vs. 44.7%, *p* < 0.001). Atherectomy (63.5% vs. 45.0%, *p* < 0.001) and drug-coated balloons (DCB; 4.7% vs. 1.7%, *p* < 0.001) were more frequently used in ISR lesions. The distal embolization rate was higher in ISR lesions (2.4% vs. 0.9%, *p*=0.02). Repeat revascularization (21.5% vs. 16.7%, *p*=0.04; Figure) was higher and freedom from MALE at 1 year was significantly lower (87% vs. 92.5%, *p* < 0.001) in the ISR group.

**Conclusion:**

Atherectomy and DCB are more frequently used to treat FP ISR lesions. Patients with FP ISR have more intraprocedural distal embolization, higher repeat revascularization procedures, and lower freedom from MALE at 1 year.

## 1. Introduction

Endovascular treatment of symptomatic PAD has emerged as the preferred revascularization modality with the advent of newer technology and combination therapies [1]. Treatment of femoropopliteal (FP) disease is a unique challenge as anatomical and mechanical forces in this vascular bed promote the development of atherosclerosis and in-stent restenosis (ISR; [2]). FP ISR is a pervasive complication with an incidence reaching 40% after 1 year of intervention and 60% at 3-years [3, 4]. Stenting and balloon angioplasty are the current mainstays of treatment, but long-term durability is poor. Treatment failure remains elevated with stent fracture and recurrent non-drug-coated stent restenosis limiting long-term patency [3]. Therefore, examining “real-world” data on the treatment and outcomes of FP ISR is highly pertinent as the optimal approach to ISR lesions remains elusive.

This study utilizes patients enrolled in the Excellence in Peripheral Artery Disease (XLPAD) registry to compare clinical outcomes of patients with symptomatic PAD undergoing endovascular treatment for FP ISR and non-ISR lesions. The primary endpoint included major adverse limb events (MALE) at 1 year, a composite of all-cause death, target limb repeat revascularization, or major amputation.

## 2. Materials and Methods

### 2.1. Study Design and Population

The XLPAD registry is an ongoing, multicenter registry of patients undergoing endovascular revascularization for infrainguinal PAD. It utilizes the REDCap web-based data acquisition system to gather data across 23 hospital sites in the United States with a core-lab adjudicated angiographic review of enrolled patients. We analyzed data of 2,895 patients undergoing endovascular treatment for symptomatic PAD with and without FP ISR between 2006 and 2019. The ISR group included first time occurrence of ISR and both drug-eluting stent (DES) and bare metal stent (BMS) ISR. Eligibility criteria for this study were adult patients undergoing percutaneous intervention for FP PAD. Patient demographics, clinical characteristics, and medications were obtained from the review of the electronic medical record. Percutaneous intervention performed was at the operator's discretion. Lesion characteristics, procedural details, and device therapies used were obtained from procedure notes. Six and 12-month outcomes after the index procedure were collected during follow-up visits. Management and clinical decisions were at the discretion of the care team.

The study was conducted in accordance with the Declaration of Helsinki and approved by the Institutional Review Board of participating hospitals. Patients from the prospective cohort were enrolled and consented prior to the index procedure. The Institutional Review Boards of the participating hospitals waived the requirement for informed consent for the retrospective cohort. Deidentified procedural and angiographic details were independently verified by the Veterans Affairs North Texas Angiography and Ultrasound core laboratory. The clinical coordinating center and data servers are located at the University of Texas Southwestern Medical Center, Dallas, TX. Periodic data audits as well as remote and on-site monitoring were performed.

### 2.2. Definitions and Outcome Measures

The study population was divided into two groups based on the presence of restenosis in the target stented lesion. ISR was defined as >50% diameter stenosis by contrast angiography or, if available, by >100% increase in the peak systolic velocity over the proximal segment or >400 cm per second via duplex ultrasound. Baseline, target lesion, and procedural characteristics were described for each group. Baseline characteristics included demographics, comorbidities, and Rutherford classification (RC): intermittent claudication (IC; RC 1–3) and critical limb ischemia (CLI; RC 4–6). Target lesion characteristics included lesion length and presence of severe calcification (at least 5 mm of calcification on both sides of the vessel), chronic total occlusion (CTO), diffuse disease (angiographic disease >30% diameter stenosis for at least 20 mm in length), and number of runoff vessels. Procedural characteristics included type and number of balloons or stents and use of debulking or CTO crossing devices (CCD). Bail out stenting was defined as operator guided stenting of the target lesion when an initial non-stent-based strategy was unsuccessful.

We examined the following outcomes: procedural success, periprocedural complications, and 1-year major adverse limb events (MALE), and major adverse cardiovascular events (MACE). Procedural success was defined as ≤ 30% residual stenosis without complication after index intervention. Periprocedural complications (immediately following the index procedure to 30 days post-procedure) included residual dissection (flow or nonflow limiting), access site hematoma (>5 cm or <5 cm), retroperitoneal hematoma, distal embolization, bleeding diathesis, acute renal failure, perforation, emergency surgery, or their composite. MALE was defined as all-cause death, repeat revascularization (endovascular or surgical), or major amputation. MACE consisted of death, myocardial infarction (MI), or stroke. The individual components of MACE and MALE were also studied.

### 2.3. Statistical Analysis

To compare baseline and lesion characteristics, procedure details, and procedure outcomes, univariate analysis was used. Continuous variables were described using mean ± standard deviation. Discrete and categorical variables were presented as numbers and percentages. Differences between the FP ISR and FP non-ISR groups were compared using either the *t*-test or the Wilcoxon rank sum test. Survival curves on the two study groups, with or without FP ISR, were generated by the Kaplan–Meier method and compared using the log-rank test. A two-tailed *p* value <0.05 was considered statistically significant. All statistical analyses were performed using SAS version 9.4 (SAS Institute Inc.) and *R* version 3.6.1 (the *R* Foundation for Statistical Computing).

## 3. Results

Of the 2,895 patients studied, 12% (*n* = 347) were treated for FP ISR. Baseline characteristics are shown in Table 1. The ISR group had a higher proportion of female patients (31.7% vs. 25.5%, *p*=0.02) and a lower prevalence of heart failure history (9.51% vs. 13.6%, *p*=0.04). A larger proportion of ISR patients received guideline directed medical therapy (GDMT): antiplatelet (single or multiple types; 94.5% vs. 89.4%, *p*=0.007) and statin (68.9% vs. 60.9%, *p*=0.003). However, overall use of optimal medical therapy across both groups was low, particularly statin therapy. A similar number of patients were on angiotensin converting enzyme inhibitor (ACEi)/angiotensin receptor blocker (ARB) therapy (57.0% vs. 61.9%, *p*=0.117). There was not a significant difference in other baseline characteristics such as age (67.2 ± 10.3 vs. 66.5 ± 9.8, *p*=0.282), other comorbidities, or smoking history (Table 1). RC : IC (63.4% vs. 63.4%, *p*=1.0), CLI (36.6% vs. 36.6%, *p*=1.0) and incidence of ankle-brachial index <0.9 (ABI; 87.1% vs. 87.9%, *p*=0.56) were similar between the ISR and non-ISR groups.

Table 2 displays target lesion characteristics. Lesion length was similar (ISR: 145 ± 99 mm vs. non-ISR: 142 ± 99 mm, *p*=0.55). However, fewer ISR lesions were severely calcified (22.4% vs. 44.7%, *p* < 0.001), CTOs (47.3% vs. 53.7%, *p*=0.02), or with diffuse disease (55.4% vs. 67.0%, *p* < 0.001). A significantly, larger proportion of ISR lesions displayed 0-1 vessel runoff (33.6% vs. 25.4%, *p* < 0.009).

Drug-coated balloons (DCBs; 4.63% vs. 1.72%, *p* < 0.001) were more frequently employed to treat ISR lesions (Table 3). A higher mean number of balloons (2.25 ± 1.75 vs. 1.96 ± 1.38,*p* < 0.009), conventional or drug-coated, were utilized and tended to have a larger mean length (102 ± 51.8 vs. 93.6 ± 52.0 mm, *p*=0.001). The use of drug-eluting stents (DES; 10.9% vs. 6.12%, *p*=0.001) was higher in the FP ISR lesion group although bare metal (BMS; 11.7% vs. 26.0%, *p*=0.001) and vascular mimetic stents (4.08% vs. 8.24%, *p*=0.007) were not as frequently deployed. On average, these stents were shorter (80.8 ± 42.8 vs. 95.8 ± 39.0 mm, *p*=0.001), and a fewer number of stents (0.82 ± 1.26 vs. 1.06 ± 1.27, *p*=0.001) were used. Debulking (63.5% vs. 44.9%, *p* < 0.001) was significantly higher for ISR lesions; however, use of CCD (37.0% vs. 49.8%, *p* < 0.001) was less common. Between the two groups, there were similar rates of bail out stenting (6.22% vs. 6.54%, *p*=0.9). Fluoroscopy time in the FP ISR and non-ISR groups was also similar (31.5 ± 18.6 vs. 32.5 ± 20.0, *p*=0.55).

**Figure 1:**
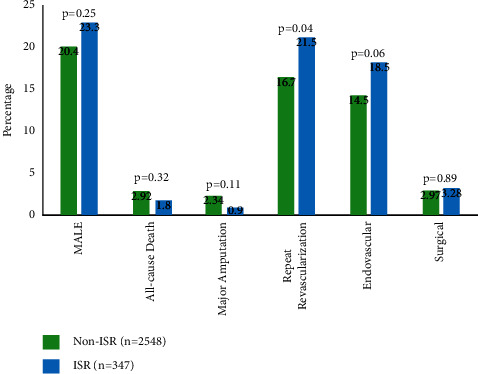
1-year major adverse limb events (MALE) and repeat revascularization for femoropopliteal (FP) non-in-stent restenosis and in-stent restenosis (ISR) groups.

**Figure 2:**
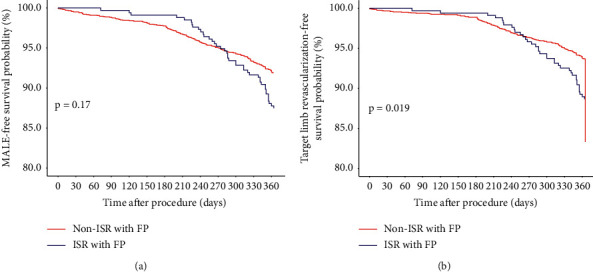
Survival curves comparing patients with femoropopliteal (FP) opliteal in-stent restenosis (ISR) and without ISR receiving endovascular treatment for symptomatic peripheral arterial disease (PAD). Kaplan–Meier curves showing that patients with FP ISR did not have significantly lower freedom from MALE at 12 months (a). Survival from target limb revascularization was significantly lower in the FP ISR group (b).

Procedural outcomes are displayed in Table 4. Comparison of 1-year outcomes is shown in Table 5 and Figure 1. Procedural success for FP ISR and FP non-ISR lesions was similar (94.3% vs. 93.0%, *p*=0.41). Even though the incidence of composite procedural complications was similar (6.78% vs. 5.97%, *p*=0.62), the rate of distal embolization (2.43% vs. 0.94%, *p*=0.02) was significantly higher in the ISR group. For 1-year outcomes, there was no difference in MACE (3.88% vs. 4.50%, *p*=0.71) or MALE (23.3% vs. 20.4%, *p*=0.25) with the *t*-test. Kaplan–Meier analysis demonstrated lower survival from MALE (87% vs. 92.5%, *p*=0.17) and target limb revascularization (TLR; 88.5% vs. 94%, *p*=0.019) at 12 months (Figure 2). Repeat revascularization (21.5% vs. 16.7%, *p*=0.04; Figure 1) was higher in the ISR group.

## 4. Discussion

This analysis from the XLPAD registry demonstrates that patients with FP ISR experience more distal embolization, higher revascularization procedures, and lower freedom from MALE at 1 year. Operators more frequently utilized drug-coated stents, atherectomy devices, and DCBs to address ISR lesions whereas the use of bare metal stents and CCDs was less common. Current data focuses on the treatment of ISR and delineating the outcomes and durability of treatment strategies. This study is the first, large, multicenter study comparing clinical outcomes of patients with and without FP ISR who undergo endovascular treatment for symptomatic PAD. Additionally, it provides pertinent details on the endovascular strategies used to treat this population.

Our findings are consistent with previously published literature of smaller sample sizes which describe higher risk of distal embolization for complex native lesions, CTOs, and ISR undergoing percutaneous treatment. Engaging the lipid and thrombotic material in the central core generates downstream debris [5]. Shammas et al. demonstrated that clinically significant distal embolization occurs in 2.4% of patients undergoing peripheral endovascular interventions. Trans-Atlantic Inter-Society Consensus (TASC) C/D lesions (OR 4.31, 95% CI 1.24–15.03; *p*=0.022) and more angiographic thrombus (OR 5.02, 95% CI 1.53–16.42; *p*=0.008) were independent predictors of distal embolization [6].

In our analysis, a debulking strategy was more frequently used by operators to treat ISR lesions. However, the use of atherectomy devices may increase the risk for distal embolization [7–9]. Mechanical disruption of ISR lesions can generate downstream debris and potential embolic material. However, multiple studies support the use of atherectomy devices for ISR treatment with favorable rates of procedural success and freedom from target lesion revascularization at 1 year [10–13]. Debulking is a widely accepted approach for treating ISR lesions as reducing smooth muscle proliferation theoretically avoids the need for repeat revascularization and restenting. This combined with distal embolization protection and antirestenotic measures has been described as a successful strategy to treat ISR [14]. Implementation of a debulking strategy is further supported by the larger proportion of 0-1 run-off vessels in the FP ISR group. With poor outflow, there is increased concern that stenting may lead to increased risk of occlusion and stent thrombosis [15].

Our study is unique as it utilizes a core-lab adjudicated angiographic review of registry patients. Fewer lesions treated for FP ISR were severely calcified, CTOs, or with diffuse disease, and there was a larger proportion with 0 to 1 runoff vessels. Known predictors of distal embolization are diffuse and/or long lesions, CTOs, and severe calcification. These parameters were not observed within the ISR group; however, distal embolization remained significantly elevated. ISR segments may have the propensity for distal embolization due to poor outflow and high utilization of atherectomy devices by operators. We suggest that ISR can be considered as a novel risk factor for distal embolization.

Within our registry, the use of DCBs (4.63% vs. 1.72%, *p* < 0.001) and DES (10.9% vs. 6.12%, *p* < 0.001) was significantly greater in the FP ISR group. This is supported by the current literature as DCBs are the most extensively investigated treatment device for ISR. Multiple randomized controlled trials display superior patency rates and freedom from target lesion revascularization at 1 year compared to percutaneous transluminal angioplasty (PTA; [16–19]). According to the 2017 European Society of Cardiology Guidelines, there is a Class IIb recommendation with Level B evidence for the use of DCBs in the treatment of ISR [20].

DES provide direct delivery of pharmacologic agents to the site of vessel injury to prevent neointimal hyperplasia and smooth muscle cell proliferation reducing the risk of restenosis [21, 22]. A limited number of prior studies have demonstrated the safety and effectiveness of DES in the treatment of FP ISR. Zeller et al. demonstrated that paclitaxel-eluting stents achieved favorable rates of primary patency (95.7% at 6-months and 78.8% at 1 year) and freedom from target lesion revascularization (96.2% at 6-months, 81.0% at 1 year, and 60.8% at 2 years). Additionally, improvements in ABI and RC were observed at 2-year follow-up (mean ABI preprocedure: 0.60 ± 0.28 vs. mean ABI at 2-years: 0.84 ± 0.22; median RC preprocedure: 3 vs median RC at 2-years: 1; [23]). A subgroup analysis comparing the use of paclitaxel-eluting stents to PTA to treat FP ISR exhibited lower rates of restenosis (44.1% vs. 90.3%, p < 0.001) and MALE (25.5% vs. 53.6%, *p* < 0.00). The use of DES was independently associated with a reduced risk of recurrent restenosis at 1 year (OR 0.2, 95% CI 0.1 to 0.6, *p*=0.006; [24]). A postmarket surveillance study on the use of the Zilver PTX stent (Cook Medical, Bloomington, IN) in treating FP ISR suggested a 5-year freedom from TLR of 73.4% [25].

BMS were infrequently used (11.7% vs. 26.0%, *p*=0.001) in the ISR group. Current evidence suggests that BMS do not provide superior patency rates at 1 year compared to PTA and carry an increased risk of restenosis [12, 26, 27]. It would be disadvantageous to use a BMS in a lesion that is already at-risk for further restenosis. Our study provides real-world evidence that operators preferentially use atherectomy and drug-coated devices to address FP ISR lesions. This is supported by the limited, but existing literature on endovascular strategies for FP PAD and ISR.

In our cohort, patients with FP ISR underwent significantly higher rates of repeat revascularization (21.5% vs. 16.7%, *p*=0.04; Figure 1). With Kaplan–Meier analysis, survival from MALE at 12 months did not reach statistical significance (*p*=0.17); however, freedom from target limb revascularization did (*p*=0.019; Figure 2). The ISR group initially had superior MALE and TLR-free survival, but at 8 months or 240 days, a crossover occurs with the non-ISR group achieving higher survival rates. DCBs and DES were preferentially used in the ISR group. This study suggests that these devices may carry short-term clinical benefits due to the drug-eluting/coated technology; however, it did not translate into long-term patency in our ISR population. This may explain the crossover in the Kaplan–Meier curves, with FP non-ISR lesions having a higher percentage of MALE-free survival and target limb revascularization-free survival beyond 8 months. As MALE was not statistically significant with both the survival model and chi-squared testing, propensity score matching was not performed.

Treatment for ISR remains an active field of investigation. Despite acute procedural success, prior studies demonstrate poor patency rates and high rates of target vessel and lesion revascularization [28, 29]. Several factors contribute to the unsatisfactory long-term outcomes. ISR is predominately driven by smooth muscle cell proliferation [30]. Intervention at an existing site of vessel injury creates a local inflammatory response that promotes neointimal hyperplasia [31]. Additionally, FP stents are at further risk of restenosis given the mechanical stressors of elongation, torsion, flexion, and extension that are unique and inherent to this vascular bed [32]. A lesion predisposed to inflammation, injury, and smooth muscle cell hypertrophy is subject to a high likelihood of revascularization after index intervention. Our incidence of revascularization (21.5% for ISR lesions) is similar when compared to prior studies. Listro et al. had a target lesion revascularization rate of 13.6% at 1 year in the DCB group and 31% in the PTA group [33]. Dippel et al. demonstrated a similar incidence of 26.5% in the laser atherectomy with PTA at 6-month follow-up [12].

Despite being performed at experienced centers and the use of novel treatment strategies, revascularization rates remain elevated and the optimal treatment modality for FP ISR is yet to be determined. Promising results from Kokkinidis et al. demonstrated that combination therapy of laser atherectomy with DCB yielded superior rates of freedom from TLR at 1 year (72.5% vs. 50.5%, *p*=0.043) and its use was associated with reduced risk of reocclusion (HR 0.08, 95% CI 0.17 to 0.38; *p*=0.002) when compared to laser atherectomy with PTA in the treatment of complex FP ISR lesions [34]. Future studies comparing clinical outcomes of patients with FP ISR undergoing endovascular monotherapy and varying combination therapies (laser atherectomy with DES or DCB, excisional atherectomy with DES or DCB) would be of interest.

A higher proportion of FP ISR patients in our study were on GDMT, antiplatelet (94.5% vs. 89.4%, *p*=0.007) and statin therapy (68.9% vs. 60.3%, *p*=0.003), and female. However, a large proportion of the non-ISR group did not receive optimal medical therapy. It has been demonstrated that a disproportionate number of PAD patients are not prescribed GDMT. In the Reduction of Atherothrombosis for Continued Health registry, out of 8273 PAD patients, only 70% received lipid-lowering therapy and only 82% received antiplatelet therapy [35]. Factors that may contribute to under-prescription include African-American race, male gender, care by nonvascular specialists, and claudicants with normal ABI's [36]. Female gender has been identified as an independent risk factor for the development of ISR and is associated with a higher prevalence of CLI, poor stent patency, and frequent restenosis in TASC C/D lesions [37]. Further research and efforts are needed to bridge the disparity observed in PAD population and specifically, female patients with PAD and restenosis.

There are important limitations to note. Our study is a retrospective observational study and investigators are responsible for enrolling patients undergoing endovascular treatment for symptomatic PAD. Therefore, it is subject to recall and selection bias and cannot account for unmeasured confounders. Conclusions must be considered as hypothesis-generating and do not determine causation. Data on the use of distal embolic filters or rate of stent fractures are not recorded and are not reflected in our analysis of periprocedural and 1-year outcomes.

## 5. Conclusion

Our large, multicenter registry demonstrates that atherectomy and drug-coated devices were more frequently used to treat FP ISR lesions. Compared to patients without ISR, patients with FP ISR experience significantly higher revascularization procedures and lower freedom from MALE at 1 year. Further studies evaluating more refined strategies to address FP PAD and ISR are needed to improve long-term patency and freedom from revascularization.

## Figures and Tables

**Table 1:** Baseline characteristics for femoropopliteal (FP) non-in-stent restenosis and in-stent restenosis (ISR) groups.

	Non-ISR (n = 2548)		ISR (n = 347)>		*P* value
Age (years)	66.5 ± 9.8		67.2 ± 10.3		0.28^*∗*^
Gender					
Male	1895	74.5%	237	68.30%	0.02
Female	649	25.5%	110	31.70%	
Race					
Caucasian (not Hispanic)	1809	73.0%	252	74.8%	0.120
African-American	435	17.5%	57	16.9%	
Hispanic	202	8.15%	28	8.31%	
Others (Asian, Native American, others)	34	1.37%	0	0.00%	
Smoking					
Within the past year	1248	49.6%	153	44.7%	0.24
>1 year ago	906	36.0%	136	39.8%	
Never	364	14.5%	53	15.50%	
Hypertension	2303	90.9%	313	90.7%	0.99
Diabetes mellitus	1368	54.4%	177	51.9%	0.41
Hyperlipidemia	2151	85.6%	299	87.9%	0.27
Chronic kidney disease	359	14.1%	42	12.10%	0.36
Coronary artery disease	1465	57.50%	198	57.1%	0.92
Heart failure	346	13.6%	33	9.51%	0.04
Prior nonfatal myocardial infarction	560	22.0%	62	17.9%	0.09
Prior stroke	209	8.2%	26	7.49%	0.73
Ankle-brachial index					
ABI <0.9	1504	87.90%	195	87.1%	0.56
0.9 ≤ ABI <1.4	187	10.9%	28	12.50%	
1.4 ≤ ABI	20	1.17%	1	0.45%	
Rutherford class					
CLI	933	36.6%	127	36.60%	1.000
IC	1615	63.4%	220	63.40%	
Antiplatelet therapy	1852	89.4%	294	94.5%	0.01
Anticoagulation therapy	191	9.60%	36	11.9%	0.25
Statin	1537	60.3%	239	68.9%	0.003
ACEi/ARB	1265	61.9%	172	57.0%	0.12

**Table 2:** Target lesion characteristics for femoropopliteal (FP) non-in-stent restenosis and in-stent restenosis (ISR) groups.

		Non-ISR (n = 3288)		ISR (n = 370)		*P* value
Lesion length (mm)		142.0 ± 99.5		145 ± 99.1		0.55^*∗*^
Severe calcification (heavily calcified)		1470	44.7%	83	22.4%	<0.001
Diffuse disease		2204	67.0%	205	55.4%	<0.001
Chronic total occlusion		1770	53.7%	175	47.3%	0.02
Run-off vessels	0–1	568	25.40%	102	33.6%	0.01
	2	697	31.2%	81	26.6%	
	3	971	43.4%	121	39.80%	

**Table 3:** Endovascular treatment strategy for femoropopliteal (FP) non-in-stent restenosis and in-stent restenosis (ISR) groups.

		Non-ISR (n = 3288)	%	ISR (n = 370)	%	*P* value
Conventional balloon		843	26.1	111	30.4	0.09
Drug-coated balloon		56	1.72	17	4.63	<0.001
Balloon length (mm)		93.60 ± 52		102 ± 51.8		0.001
Number of balloons	0	238	7.32	30	8.17	0.05
	1	121	37.2	113	30.8	
	≥2	1800	55.5	224	61.0	
Number of balloons, continuous		1.96 ± 1.38		2.25 ± 1.75		0.009^*∗*^
Bare-mental stent		845	26.0	43	11.7	<0.001
Drug-eluting stent		199	6.12	40	10.9	<0.001
Covered stent only		93	2.86	18	4.89	0.05
Vascular mimetic stent		268	8.24	15	4.08	0.007
Stent length (mm)		95.8 ± 39		80.8 ± 42.8		<0.001
Number of stents, categorical	0	1480	45.4	214	58.1	<0.001
	1	801	24.6	76	20.7	
	≥2	974	30.0	78	21.20	
Number of stents, continuous		1.06 ± 1.27		0.82 ± 1.26		<0.001
Bail out stenting		215	6.54	23	6.22	0.9
Debulking device		1480	44.9	235	63.5	<0.001^*∗*^
CTO crossing devices		1640	49.8	137	37.0	<0.001
Fluoroscopy time (min)		32.5 ± 20		31.53 ± 18.6		0.55^*∗*^

**Table 4:** Procedural outcomes for femoropopliteal (FP) non-in-stent restenosis and in-stent restenosis (ISR) groups.

	Non-ISR (n = 3288)	%	ISR (n = 370)	%	*P* value
Procedural success	3050	93.0	348	94.3	0.41
Procedural complications	196	5.97	25	6.78	0.62
Residual dissection (flow-limiting)	41	1.25	6	1.62	0.47
Residual dissection (nonflow limiting)	56	1.70	4	1.08	0.52
Access-site hematoma < 5 cm	7	0.21	1	0.27	0.58
Access-site hematoma > 5 cm	12	0.37	0	0.00	0.62
Retroperitoneal hematoma	11	0.33	0	0.00	0.62
Distal embolization	31	0.94	9	2.43	0.02
Bleeding diathesis	4	0.12	1	0.27	0.41
Acute renal failure	5	0.15	1	0.27	0.47
Perforation	17	0.52	2	0.54	1.00
Emergency surgery	5	0.15	0	0.00	1.00
Composite hematoma (access site <5 cm, >5 cm, retroperitoneal)	30	0.91	1	0.27	0.362

**Table 5:** 1-year outcomes for femoropopliteal (FP) non-in-stent restenosis and in-stent restenosis (ISR) groups.

	Non-ISR (n = 2548)	%	ISR (n = 347)	%	P value
Death	70	2.92	6	1.80	0.32
Repeat endovascular intervention	346	14.5	62	18.5	0.06
Surgical target limb revascularization	71	2.97	11	3.28	0.89
Amputation in target limb (major)	56	2.34	3	0.90	0.11
MI	41	1.72	5	1.50	1.00
Stroke	10	0.42	2	0.60	0.65
Major adverse limb eventsa	490	20.4	78	23.3	0.25
Major adverse cardiovascular eventsb	108	4.50	13	3.88	0.71
Repeat revascularizationc	399	16.7	72	21.5	0.04

## Data Availability

Due to the nature of this research, participants of this study did not agree for their data to be shared publicly, so supporting data are not available. The data are stored within the XLPAD registry where IRB approval is required for researchers and clinicians to gain access to registry for data analysis and entry.

## Abbreviations

mm: millimeter

## References

[1] Lin J. H., Brunson A., Romano P. S., Mell M. W., Humphries M. D. (2019). Endovascular-first treatment is associated with improved amputation-free survival in patients with critical limb ischemia. *Circulation: Cardiovascular Quality and Outcomes*.

[2] Cheng C. P., Wilson N. M., Hallett R. L., Herfkens R. J., Taylor C. A. (2006). In Vivo MR angiographic quantification of axial and twisting deformations of the superficial femoral artery resulting from maximum hip and knee flexion. *Journal of Vascular and Interventional Radiology*.

[3] Ho K. J., Owens C. D. (2017). Diagnosis, classification, and treatment of femoropopliteal artery in-stent restenosis. *Journal of Vascular Surgery*.

[4] Singh G. D., Armstrong E. J., Laird J. R. (2014). Femoropopliteal in-stent restenosis: current treatment strategies. *The Journal of Cardiovascular Surgery*.

[5] Zankar A., Brilakis E., Banerjee S. (2011). Use of embolic capture angioplasty for the treatment of occluded superficial femoral artery segments. *The Journal of invasive cardiology*.

[6] Shammas N. W., Shammas G. A., Dippel E. J., Jerin M., Shammas W. J. (2009). Predictors of distal embolization in peripheral percutaneous interventions: a report from a large peripheral vascular registry. *The Journal of invasive cardiology*.

[7] Kasirajan K., Haskal Z. J., Ouriel K. (2001). The use of mechanical thrombectomy devices in the management of acute peripheral arterial occlusive disease. *Journal of Vascular and Interventional Radiology*.

[8] Roberts D., Niazi K., Miller W. (2014). Effective endovascular treatment of calcified femoropopliteal disease with directional atherectomy and distal embolic protection: Final results of the DEFINITIVE Ca++trial. *Catheterization and Cardiovascular Interventions*.

[9] Lam R. C., Shah S., Faries P. L., McKinsey J. F., Kent K. C., Morrissey N. J. (2007). Incidence and clinical significance of distal embolization during percutaneous interventions involving the superficial femoral artery. *Journal of Vascular Surgery*.

[10] Shammas N. W., Jones-Miller S., Shammas G. A., Shammas W. J. (2020). Jetstream atherectomy in treating femoropopliteal in-stent restenosis: meta-analysis of the JETSTREAM-ISR and JET-ISR trials. *The Journal of invasive cardiology*.

[11] Schmidt A., Zeller T., Sievert H. (2014). Photoablation Using theTurbo-Booster andExcimer Laser for In-Stent RestenosisTreatment: Twelve-Month Results From the PATENT Study. *Journal of Endovascular Therapy*.

[12] Dippel E. J., Makam P., Kovach R. (2015). Randomized Controlled Study of Excimer Laser Atherectomy for Treatment of Femoropopliteal In-Stent Restenosis. *JACC: Cardiovascular Interventions*.

[13] Palena L. M., Manzi M. (2012). Extreme below-the-knee interventions: retrograde transmetatarsal or transplantar arch access for foot salvage in challenging cases of critical limb ischemia. *Journal of Endovascular Therapy*.

[14] Shammas N. (2013). An overview of optimal endovascular strategy in treating the femoropopliteal artery: mechanical, biological, and procedural factors. *International Journal of Angiology*.

[15] Kinlay S. (2016). Management of critical limb ischemia. *Circulation: Cardiovascular Interventions*.

[16] Krankenberg H., Tübler T., Ingwersen M. (2015). Drug-coated balloon versus standard balloon for superficial femoral artery in-stent restenosis. *Circulation*.

[17] Ott I., Cassese S., Groha P. (2017). ISAR‐PEBIS (Paclitaxel‐Eluting Balloon Versus Conventional Balloon Angioplasty for In‐Stent Restenosis of Superficial Femoral Artery): A Randomized Trial. *Journal of the American Heart Association*.

[18] Kinstner C. M., Lammer J., Willfort-Ehringer A. (2016). Paclitaxel-Eluting Balloon Versus Standard Balloon Angioplasty in In-Stent Restenosis of the Superficial Femoral and Proximal Popliteal Artery. *JACC: Cardiovascular Interventions*.

[19] Cassese S., Wolf F., Ingwersen M. (2018). Drug-coated balloon angioplasty for femoropopliteal in-stent restenosis. *Circulation: Cardiovascular Interventions*.

[20] (2021). ESC guidelines on the diagnosis and treatment of peripheral arterial diseases, in collaboration with the European society for vascular surgery (ESVS) | European heart journal | oxford academic. https://academic.oup.com/eurheartj/article/39/9/763/4095038.

[21] James S. K., Stenestrand U., Lindbäck J. (2009). Long-term safety and efficacy of drug-eluting versus bare-metal stents in Sweden. *New England Journal of Medicine*.

[22] Lee C.-H., Yu C.-Y., Chang S.-H. (2014). Promoting endothelial recovery and reducing neointimal hyperplasia using sequential-like release of acetylsalicylic acid and paclitaxel-loaded biodegradable stents. *International Journal of Nanomedicine*.

[23] Zeller T., Dake M. D., Tepe G. (2013). Treatment of femoropopliteal in-stent restenosis with paclitaxel-eluting stents. *JACC: Cardiovascular Interventions*.

[24] Murata N., Takahara M., Soga Y. (2016). Drug-Eluting Stent vs Percutaneous Transluminal Angioplasty for Treatment of Femoropopliteal In-Stent Restenosis. *Journal of Endovascular Therapy*.

[25] Sugimoto M., Komori K., Yokoi H. (2021). Long-term effectiveness of a drug-eluting stent for femoropopliteal in-stent restenosis: subanalysis of the zilver PTX Japan post-market surveillance study. *Journal of Endovascular Therapy*.

[26] Becquemin J.-P., Favre J.-P., Marzelle J., Nemoz C., Corsin C., Leizorovicz A. (2003). Systematic versus selective stent placement after superficial femoral artery balloon angioplasty: a multicenter prospective randomized study. *Journal of Vascular Surgery*.

[27] Zdanowski Z., Albrechtsson U., Lundin A. (1999). Percutaneous transluminal angioplasty with or without stenting for femoropopliteal occlusions? A randomized controlled study. *International Angiology*.

[28] Laird J. R. (2006). Limitations of percutaneous transluminal angioplasty and stenting for the treatment of disease of the superficial femoral and popliteal arteries. *Journal of Endovascular Therapy*.

[29] Kandarpa K., Becker G. J., Hunink M. G. M. (2001). Transcatheter interventions for the treatment of peripheral atherosclerotic lesions: part I. *Journal of Vascular and Interventional Radiology*.

[30] Yeo K.-K., Malik U., Laird J. R. (2011). Outcomes following treatment of femoropopliteal in-stent restenosis: a single center experience. *Catheterization and Cardiovascular Interventions*.

[31] Hoffmann R., Mintz G. S., Dussaillant G. R. (1996). Patterns and Mechanisms of In-Stent Restenosis. *Circulation*.

[32] Kim W., Choi D. (2018). Treatment of femoropopliteal artery in-stent restenosis. *Korean Circulation Journal*.

[33] Liistro F., Angioli P., Porto I. (2014). Paclitaxel-eluting balloon vs. standard angioplasty to reduce recurrent restenosis in diabetic patients with in-stent restenosis of the superficial femoral and proximal popliteal arteries: the DEBATE-ISR study. *Journal of Endovascular Therapy*.

[34] Kokkinidis D. G., Hossain P., Jawaid O. (2018). Laser atherectomy combined with drug-coated balloon angioplasty is associated with improved 1-year outcomes for treatment of femoropopliteal in-stent restenosis. *Journal of Endovascular Therapy*.

[35] Bhatt D. L., Steg P. G., Ohman E. M. (2006). International prevalence, recognition, and treatment of cardiovascular risk factors in outpatients with atherothrombosis. *JAMA*.

[36] Gober L, Bui A, Ruddy JM (2020). Racial and gender disparity in achieving optimal medical therapy for inpatients with peripheral artery disease. *Annals of vascular medicine and research*.

[37] Stavroulakis K., Donas K. P., Torsello G., Osada N., Schönefeld E. (2015). Gender-related long-term outcome of primary femoropopliteal stent placement for peripheral artery disease. *Journal of Endovascular Therapy*.

